# Supplementary material for: Ecological changes over 90 years at Low Isles on the Great Barrier Reef
Source: Nat Commun. 2019 Sep 27;10:4409. doi: 10.1038/s41467-019-12431-y (PMC6765017; doi:10.1038/s41467-019-12431-y)
Supplement: Supplementary file 1 — Supplementary Information [file 41467_2019_12431_MOESM1_ESM.pdf]

Fine et al., **Ecological changes over 90 years at Low Isles on the Great Barrier Reef**

Supplementary Information

Supplementary figures

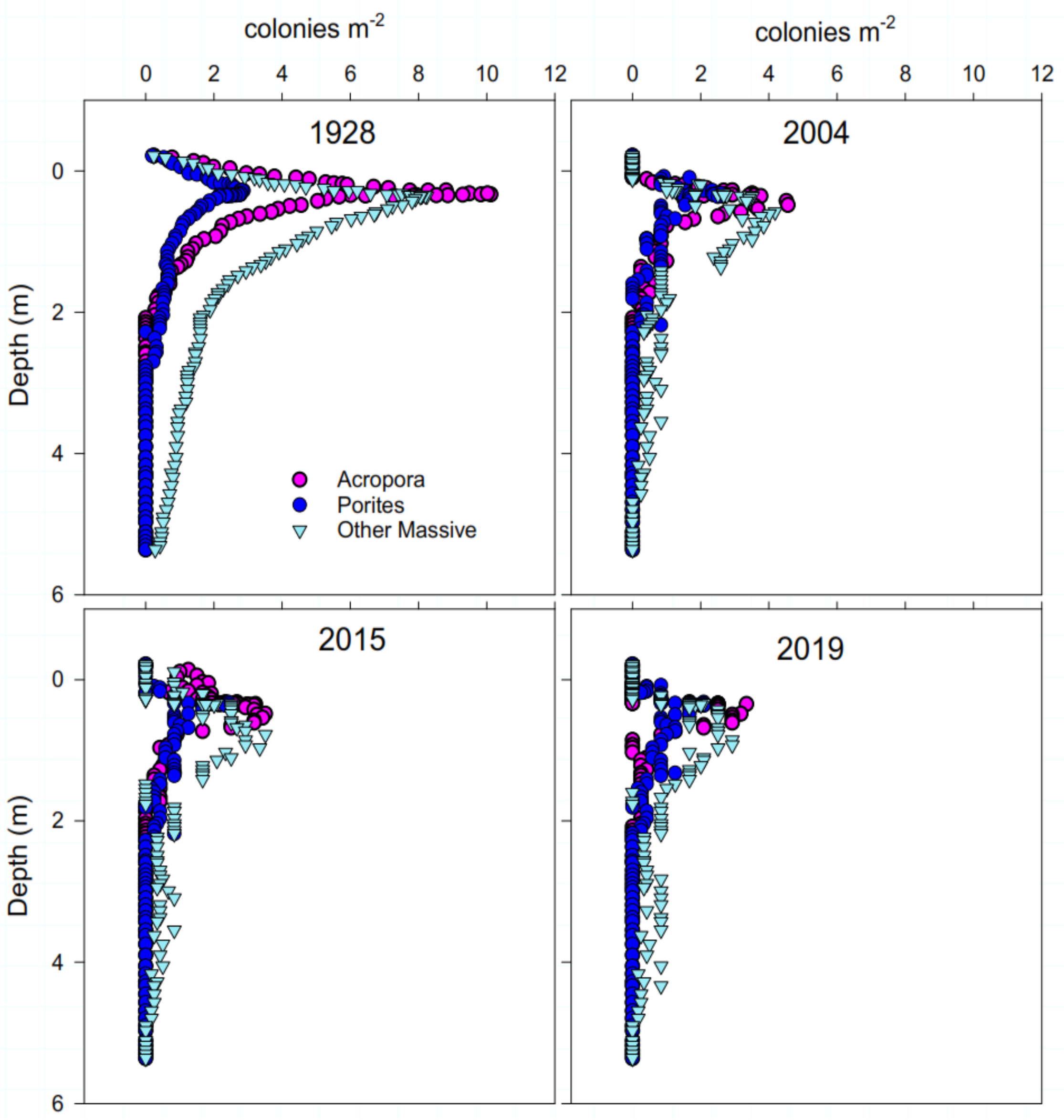

Supplementary Figure 1: **Changes in coral composition along Traverse 1 from 1928[1] -2019**, showing a decrease in the number of colonies per area over time and particularly a shift to higher homogeneity with a major decrease in the number of *Acropora* colonies.

Supplementary Table 1: Changes to intertidal habitats 1928-2019

Descriptions of mapped habitats on the 1928 GBRE map[1] which were revisited since then by a few researchers[2, 3, 4, 5] including the present study. While qualitative, these descriptions underline the changes that the reefs at LI have undergone over the past 90 years.

Location	1928-29	1945	1954	1993	2004	2015	2019
**Anchorage**	Scleractinian corals massive Porites, Favites, and Goniastrea and branching or digitate Acropora forms, dominate the patch reefs and margins of the Anchorage	Patch reefs in the "Anchorage had grown a recognizable amount since 1928"	Corals were again damaged by TC0207, live hard coral to be ''sparse,'' with dead scleractinian coral overgrown by abundant soft coral	Soft corals and benthic algae now dominate patch reefs and the margins of the Anchorage	Soft corals dominate. Inshore hard coral includes Acropora and Montipora.<br><br>The Seaward slope has large colonies of Heliopora and branching Porites	Soft corals dominate both inshore and seaward. Signs of physical destruction by Cyclone.	Inshore section has a high cover of soft corals and no hard coral. Seaward slope has some hard coral, primarily massive and encrusting. Few Acropora only.
**Porites Pond**	It has a flourishing population of coral, amongst which massive *Porites* of large size is dominant.	Coral restricted by newly developing rampart	Bottom of broken coral (mostly Acropora) with little sand, Diadema dominant: Fungia, Porites lutea		Sand and seagrass High sediment Dead large Porites	Sand and sea grass High sedimentati on Dead large Porites, in some, a living patch was observed	Sand and rubble. Dead large Porites microatolls
**Madrepore Moat**	A stretch containing a very healthy growth of coral but of rather fewer spp than in other parts.	Mostly intact	Digitate Montipora ,Acropora dominate, "Montipora lawn" nearby.		No living coral	Digitate Montipora and Acropora emerging from coral rubble	Rubble and rock, Padina very common, small 5 cm Porites, a few 10 cm Favids, Sarcophyton 5 cm, Microatoll of Porties 50 cm across, Halimeda, 2 holothurian spp common, *Montipora digitata* (5 cm) numerous, 30 cm Tridacna *T.gigas* (30 cm), Linckia
**Fungia Moat**	Three spp. At least of Fungia dominant among dead	Very greatly restricted by invasion of rampart and sand flat. Fungia	No Fungia		No living Fungia or any other spp.	No living coral	Halimeda,<br><br>Small Favids, and Porites (3 cm), Sarcophyton

	branched *Acropora*	fungites common					common;Single *Acropora millepora* (10 cm)<br><br>Holothuria and Stichopus,<br><br>*T. crocea*<br><br>*T. gigas* (80 cm) present;<br><br>No living Fungia at all.
**Western Moat**	A very considerable number of coral spp. are represented. At least fifteen genera.	Cut off by Asterina spit	Cyphastrea, Montipora and favia dominant.		Cyphastrea and Favids present	Cyphastrea and Favids present	High cover of seagrass, 80 cm high Sargassum, Porites microcolonies, two colonies of Dipsastrea (10cm), Holothuria and Stichopus,<br><br>Gobi & shrimp present; seagrass common

Supplementary Table 2: Intertidal coral and invertebrates communities 1954 and 2019

Revisiting a survey performed in 1954[6] we returned to the exact locations in 2019 and recorded coral and invertebrates species in each site.

	1954		2019	
**Site**	**Hard Coral**	**Invertebrates**	**Hard Coral**	**Invertebrates and algae**
VII (16°23'1.04"S 145°33'58.22"E) Porites pond	21 coral spp. Massive Porites dominant; Fungia sub-dominate.	5 invert spp. Diadema dominant; Synapta and Stichopus present.	No living corals, rubble only, no Porites, Fungia skeletons nearby	A single *Tridacna gigas* (30 cm).
XXVIII (16°23'28.28"S 145°33'46.23"E): Madrepore moat	18 coral spp. *Goniastrea pectinata* and *Porites lutea* co-dominate; *Montipora divercata*, *Pocillopora damicornis*, *Favia Palida* and *Favites abdita* sub-dominate.	7 invert spp. Hippopus and Stichopus common; Echinometra, Linckia and Pinctada present.	No living corals.	Sargassum (40 cm), Padina high cover, lots of turf algae. Holothuria, and *Tridacna. crocea*, common.
XVII (16°23'1.84"S 145°33'39.41"E): Northern moat	15 spp. of corals *Goniastrea pectinate* and Platygyra co-dominate.	16 invert spp. Sarcophyton co-dominant; Diadema, Echimometra, Holothuria, Synapta, Pinctada and several soft coral species present.	3 coral spp. Few 5 cm Favids.	7 invert spp. One 60 cm *T. gigas* Sarcophyton, dominant; Lobophyton sub-dominant.

XXXVII (16°23'23.06"S<br><br>145°34'16.56"E) Eastern side	13 coral spp.<br><br>*M. divercata*, *Leptastrea purpurea* co-dominant; *P. damicornis*, *F. abdita* and *Acropora pulchra* sub-dominate.<br><br>"Here Acropora colonies were flourishing and were not attached to the substratum but merely embedded in sand and rubble."	9 invert spp.<br><br>Diadema dominant; Hippopus, Sinapta present.	4 coral spp.<br><br>*Porites microatolls*, dominant; *Acropora palifera*, *Montipora digitate* co-dominant; Favites, Leptastrea, sub-dominant.	6 invert spp.<br><br>Sarcophyton sub-dominant; Stichopus, *T.gigas*, *T. crocea* common.
XXXVI (16°23'33.10"S<br><br>145°34'21.27"E) Eastern moat	11 coral spp.<br><br>*Leptastrea purpurea*, *P. lutea*, Platygyra, *Cyphastrea serailia* and *Goniastrea retiformis* sub-dominant.<br><br>"Acropora colonies flourishing"	11 invert spp.<br><br>Zoanthus dominant; Diadema and Hippopus present.	Site now rubble and mangroves	Site now rubble and mangroves
VIII (16°22'58.04"S<br><br>145°34'2.30"E) Lone mangrove	11 coral spp.<br><br>*M. divercata* and *P. damicornis* co-dominant.	8 invert spp.<br><br>Diadema dominant; Synapta, Stichopus and Cyprea present.	1 coral spp.<br><br>Dead Favids, single massive Porites (5 cm).	3 invert spp.<br><br>Sargassum (40 cm) dominant; Thalassia, *T. crocea* (10 cm) common; Holothuria present.
IX (16°22'59.23"S 145°34'9.19"E) Eastern side	26 coral spp.<br><br>*M. divercata*, *G. retiformis* and *Millepora tenera* dominant; *P. lutea*, *Acropora squamosa*, *A.*	8 coral spp.<br><br>*T. crocea*, Echinometra and Zoanthus sub-dominant; *Tridacna fossor*, Palythoa	6 coral spp.<br><br>Scattered Favids, mostly rubble, *P. damicornis* (15 cm), plating Montipora (up to 100 cm) dominant;	6 invert spp.<br><br>80 cm *T. gigas*, Sarcophyton (10-15 cm) common.

	humilis, *P. damicornis*, Montipora sp. sub-dominant.	common; Holothuria and Stichopus present.	*A. pulchra*, *M. digitate*, Fungia (5 cm) present.	
XXX (16°23'24.14"S 145°34'12.53"E) Mangrove area	11 coral spp. *Porites lobata* dominant.	4 invert spp. Hippopus sub-dominant; Diadema, Synapta and *Monetaria annularis* common.	1 coral spp. Rubble, *M. digitata* common but mostly dead	2 invert spp. Halimeda, Sargassum common; Linckia, Holothuria, present (sand 50 %).
XXII (16°23'16.70"S 145°33'40.35"E) Stony flat	9 coral spp. *P. lutea* dominant; *M. divercata* and *P. damicornis* sub-dominant.	5 invert spp. Siphonaria sp., Cham and *T. crocea* dominant; *Trochus obeliscus*, Spondylus sub-dominant.	No living coral.	2 invert spp. Padina, Thalassia, dominant; *T. gigas* (15cm), *T. crocea* common.
XXVII (16°23'18.53"S 145°33'42.70"E) Montipora lawn	9 coral spp. *"Montipora divaricata (dominant)* formed beds or "lawns" in shallow water." *"Pocillopora damicornis* was more widespread but never as dense" also sub-dominate; *G. pectinata* and *L. purpurea*.	9 invert spp. Hippopus and Stichopus common; Trochus, Echinometra, Linckia, Pinctada, present.	3 coral spp. Massive Porites (5-10 cm), Favites (5-10 cm) common; Goniastrea, Porites microatoll (80 cm), mostly dead, *M. digitata* (5 cm) present.	3 invert spp. Padina, Sargassum, *T. crocea* common; Linkia, *Sarcophyton* (10cm) present. quite a dead site.
XXXV (16°23'23.68"S 145°34'21.04"E) Eastern side	19 coral spp. *Acropora hebes*, *G. pectinate* and *M. divercata* dominant; Montipora spp., *A. pulchra*, *P. damicornis* sub dominate; *F. palida P. lutea*	19 invert spp. *M. annulus* dominant; *T. crocea*, Echinometra, Linckia, *Rochia nilotica* common.	6 coral spp. Acantastrea >1m across, Acropora, (few 10cm), *P. damicornis* (many, 15 cm +), Porites (5-20 cm many) and microatols, many dead. Sandy substrate with rubble, lots of	4 invert spp. *T. crocea*, *T. gigas* (1m), Stichopus, Linckia present.

	and Platygyra sub-doninant.		Porites bommies, a few plating Acropora (50 cm).	
XXXII (16°23'32.00" S<br><br>145°34'14.82" E) Eastern side	2 coral spp.<br><br>*P. lobata* dominant.	5 invert spp.<br><br>Anthopleura sp. and Nerita sp. Dominant; Cerithium sp. Common.	Rubble, no coral spp.	3 invert spp.<br><br>Ophurids in rubble common; Stichopus, Linckia present.

Supplementary Table 3: Stress events at Low Isles 1928-2019

Symbols (~, *) represent an event regardless of severity. Sources include the Australian Bureau of Meteorology for cyclones and floods, present study and AIMS LMTP for CoTS and bleaching events.

Year	Flood	Cyclone	CoTS	Bleaching
1934		TC0113		
1950		TC0207		
1956	~			
1972	~			
1973		Madge		
1977		Otto	*	
1990		Ivor		
1994			*	
1996	~			
1998				severe
1999		Rona	*	
2000		Steve		
2002				severe
2004	~			
2006	~			
2008	~			
2009	~			
2011	~	Yasi		
2013	~			
2014	~	Marcia		
2017		Debbie		severe
2018	~			
2019	~			local

Supplementary References

1. Manton SM, Stephenson T. *Ecological surveys of coral reefs*. *Sci. Rep. Great Barrier Reef Exped. 1928-29* **3** (1935).

2. Stephenson W, Endean R, Bennett I. An ecological survey of the marine fauna of Low Isles, Queensland. *Mar Freshwater Res* **9**, 261-318 (1958).

3. Fairbridge RW, Teichert C. The low isles of the Great Barrier Reef: a new analysis. *Geogr J* **111**, 67-88 (1948).

4. Moorhouse F. The cyclone of 1934 and its effects on Low Isles, with special observations on Porites. *Reports of the Great Barrier Reef Committee* **4**, 36-47 (1936).

5. Bell PR, Elmetri I. Ecological indicators of large-scale eutrophication in the Great Barrier Reef lagoon. Oceanogr *Lit Rev* **12**, 1145 (1995).
