# Supplementary material for: Ecological changes over 90 years at Low Isles on the Great Barrier Reef
Source: Nat Commun. 2019 Sep 27;10:4409. doi: 10.1038/s41467-019-12431-y (PMC6765017; doi:10.1038/s41467-019-12431-y)
Supplement: Supplementary file 3 — Reporting Summary [file 41467_2019_12431_MOESM3_ESM.pdf]

nature research

Corresponding author(s): Maoz Fine

Last updated by author(s): Sep 1, 2019

Reporting Summary

Nature Research wishes to improve the reproducibility of the work that we publish. This form provides structure for consistency and transparency in reporting. For further information on Nature Research policies, see Authors & Referees and the Editorial Policy Checklist.

Statistics

For all statistical analyses, confirm that the following items are present in the figure legend, table legend, main text, or Methods section.

n/a	Confirmed	
☒	☐	The exact sample size (*n*) for each experimental group/condition, given as a discrete number and unit of measurement
☒	☐	A statement on whether measurements were taken from distinct samples or whether the same sample was measured repeatedly
☒	☐	The statistical test(s) used AND whether they are one- or two-sided<br>*Only common tests should be described solely by name; describe more complex techniques in the Methods section.*
☒	☐	A description of all covariates tested
☒	☐	A description of any assumptions or corrections, such as tests of normality and adjustment for multiple comparisons
☒	☐	A full description of the statistical parameters including central tendency (e.g. means) or other basic estimates (e.g. regression coefficient) AND variation (e.g. standard deviation) or associated estimates of uncertainty (e.g. confidence intervals)
☒	☐	For null hypothesis testing, the test statistic (e.g. *F*, *t*, *r*) with confidence intervals, effect sizes, degrees of freedom and *P* value noted<br>*Give P values as exact values whenever suitable.*
☒	☐	For Bayesian analysis, information on the choice of priors and Markov chain Monte Carlo settings
☒	☐	For hierarchical and complex designs, identification of the appropriate level for tests and full reporting of outcomes
☒	☐	Estimates of effect sizes (e.g. Cohen's *d*, Pearson's *r*), indicating how they were calculated

Our web collection on statistics for biologists contains articles on many of the points above.

Software and code

Policy information about availability of computer code

Data collection	To correlate a global positioning system (GPS) with the original maps, we digitized the maps using Georeferencer (Klokan Technologies GmbH) and Google Earth.<br>In 2004, digital photos 1x1.5 m with an overlap of at least 60 cm were stitched (Adobe Photoshop) to form a continuous panoramic traverse on which a set of 1x1 m quadrate (true to scale) was electronically overlayed.<br>Coral Point Count with Excel extension (CPCE) was used to analyze coral species diversity, live cover and colony size.<br>Data from the old 1928 GBRE reports was mined using WebPlot Digitizer V.4.1.
Data analysis	MS Office, Excel 2016, Systat SigmaPlot 12.3, CPCe V4.1.

For manuscripts utilizing custom algorithms or software that are central to the research but not yet described in published literature, software must be made available to editors/reviewers. We strongly encourage code deposition in a community repository (e.g. GitHub). See the Nature Research guidelines for submitting code & software for further information.

Data

Policy information about availability of data

All manuscripts must include a data availability statement. This statement should provide the following information, where applicable:

- Accession codes, unique identifiers, or web links for publicly available datasets
- A list of figures that have associated raw data
- A description of any restrictions on data availability

Provide your data availability statement here.

Field-specific reporting

Please select the one below that is the best fit for your research. If you are not sure, read the appropriate sections before making your selection.

☐ Life sciences ☐ Behavioural & social sciences ☒ Ecological, evolutionary & environmental sciences

For a reference copy of the document with all sections, see nature.com/documents/nr-reporting-summary-flat.pdf

Ecological, evolutionary & environmental sciences study design

All studies must disclose on these points even when the disclosure is negative.

Study description	In this study we surveyed sites and locations which were first surveyed in 1928-9 and then in 1954. We repeated the very same surveys and visited selected sites in 2004, 2015 and 2019. Therefore the design is based on what the original studies in 1928 and 1954 performed. Traverses were recorded using a belt transect and the selected sites by visual surveys.
Research sample	As stated above, the study revisits the 1928-9 reports. Data was extracted from the old 1928 GBR expedition reports and compared with our data which was collected in a similar manner along the same traverses and in the same locations which were accurately mapped in the original study. We returned to exactly the same sites in 2004, 2015 and 2019.
Sampling strategy	Since the present study followed with great precision studies from 1928 and 1954, sample size, traverses length and the number of sites visited were all dictated by the original studies. We believe it represents well the changes in coral cover and community structure at Low Isles.
Data collection	The three traverses measured by the GBRE were used as permanent transects. Traverse 1 on the western side, Traverse 2 on the north western and Travers 3 in the southeastern side of the island. Intertidal sections of the traverse were performed at low tide in 2004 and 2015, at high tide in 2019. Traverses were measured using a measuring tape and a digital camera. In each site, three traverses were performed in parallel (~10 m apart) to cover well the area of the 1928 traverse from start to endpoint. The general description of the moats, ponds and intertidal sections was performed by four people (MF, EMF, SD and OHG in 2004, MF and OHG in 2015, 2019) who overlapped and repeated their visits to each site.
Timing and spatial scale	Surveys were performed in September 2004 and 2015 and April 2019. Since we examined long-lived coral populations, and long term changes to the reef community structure, timing of the visits to Low Isles was of minor importance. The original 1928 surveys were performed for a few months whereas in the present study it took a week to complete in each of the visits to the Island.
Data exclusions	No data was excluded
Reproducibility	Reproducibility in the case of the present study is our ability to return to the exact location that was surveyed in 1928 and 1954. To track the footsteps of the coral ecology work performed by the 1928 expedition and a follow-up expedition in 1954, the original GBRE map, 1: 5000-scale of Low Isles was used. To correlate a global positioning system (GPS) with the original maps, we digitized the maps using Georeferencer (Klokan Technologies GmbH) and Google Earth. The lighthouse and the base of an old jetty were used as georeferenced sites. By linking points on the image with those same locations in the geographically referenced data, a polynomial transformation is created, converting the location of the entire image to the correct geographic location. The exact location of habitats explored and reported by the 1928 GBRE and 1954 expedition were mined from the digitized map and a handheld GPS (Garmin 2004, 2015; Suunto Ambit3 in 2019) was used to return to these locations.
Randomization	Randomization in the present study was not sought. We repeated the 1928/1954 surveys, returning to exactly the same sites in every visit to the Island.
Blinding	Blinding was not relevant for this study as we selected for sites that were previously examined in 1928/1954.

Did the study involve field work? ☒ Yes ☐ No

Field work, collection and transport

Field conditions	Conditions varied between visits to the Low Isles Reef but overall, in all visits the sea was calm with temperatures ranging between 26-28 C. in March 2019 visibility was poor towards the end of our expedition but we were lucky enough to complete all the tasks before the heavy rain.
Location	Low Isles Reef is an inshore reef located in the northern Great Barrier Reef, Australia (168239 S, 1458349 E).
Access and import/export	Surveys were performed under permit No. G18/41261.1 from the Great Barrier Reef Marine Park Authority. In 2015 also collected selected coral fragments for the purpose of identification. Coral fragments were transported by plane to The University of Queensland where they were properly cleaned and identified. We plan to deposit these corals at the Natural History Museum in London, to make it available next to the corals collected in 1928.
Disturbance	Only disturbance by the study was sampling coral fragments for identification in 2015. only very few fragments were sampled. the survey and inspection of sites was non-destructive.

Reporting for specific materials, systems and methods

We require information from authors about some types of materials, experimental systems and methods used in many studies. Here, indicate whether each material, system or method listed is relevant to your study. If you are not sure if a list item applies to your research, read the appropriate section before selecting a response.

Materials & experimental systems

n/a	Involved in the study
☒	☐ Antibodies
☒	☐ Eukaryotic cell lines
☒	☐ Palaeontology
☒	☐ Animals and other organisms
☒	☐ Human research participants
☒	☐ Clinical data

Methods

n/a	Involved in the study
☒	☐ ChIP-seq
☒	☐ Flow cytometry
☒	☐ MRI-based neuroimaging
